# The views of intensive care nurses regarding short-term deployment

**DOI:** 10.4102/curationis.v38i1.1478

**Published:** 2015-10-12

**Authors:** Mokgadi C. Matlakala

**Affiliations:** 1 Department of Health Studies, University of South Africa, South Africa

## Abstract

**Background:**

Short-term deployment of nurses is usually used within the hospital units in order to ‘balance the numbers’ or to cover the shortage of staff in the different units. Often nurses in the intensive care unit (ICU) are sent to go and assist in other units, where there is not enough nursing staff or when their own unit is not busy.

**Objectives:**

The objective of this study was to explore the views of the ICU nurses regarding short-term deployment to other units.

**Method:**

A qualitative design was used, following interpretivism. The study was conducted in the ICUs of two hospitals in Gauteng Province, South Africa. Data were collected through focus group interviews with a purposive sample of registered nurses working in the selected ICUs, transcribed verbatim and analysed using open coding.

**Results:**

The participants shared a similar view that deployment to other units should be based on a formal agreement, with policies and procedures. Consultation and negotiation are recommended prior to deployment of staff. Management should recognise and acknowledge expertise of ICU nurses in their own speciality area.

**Conclusion:**

The findings call for redesign of a deployment policy that will suit nurses from the speciality areas such as ICU.

## Introduction

Short-term deployment of nurses is a temporary placement of nurses in a ward or other unit for a period of 12 hours or less. Short-term deployment of nurses is usually used within the hospital units in order to ‘balance the numbers’ or to cover the shortage of staff in the different units. Often, nurses working in the intensive care unit (ICU) are required to help in other units if there is not enough staff, or their own unit is not busy. An ICU nurse is a professional and/or registered nurse who is trained, or is experienced in caring for the critically-ill patients in the ICUs. In this study, nurses work in the ICUs where short-term deployment is practiced. Short-term deployment of nurses from ICU may be to general wards that are not exactly the same as ICU as regards general layout, speciality area, patient care and general ward routine. The aim of deployment is to cover the shortage of nurses in the particular unit for a duty shift.

The nursing profession is experiencing a shortage of nurses, which has been attributed to many factors, including nurse emigration, morbidity and retirement (Breier 2007:123). A WHO report indicated that 60% of South African institutions had trouble replacing nurses who had emigrated (Hamilton & Yau 2009). The decline in the number of nurses indicates challenges in nurse staffing in the different units (Scribante & Bhagwanjee 2007). Randa et al. (2014:61) indicate that career change also contributes to nursing shortage. Nursing shortages affect the workload of existing staff and pose a potential threat to the continuity of care and patients’ safety (Scott 2003:11). According to Ogle and Glass (2014:2), with nursing shortages attention is often focused on the quick solutions to recruit more nurses.

The South African government uses measures such as community service and occupational specific dispensation to address the shortage of health staff (Erasmus & Breier 2009:123). However, the challenge with community service nursing is the lack of expertise of the new nurses doing community service to work in the specialised areas such as ICU. In other provinces in South Africa, existing short and medium term initiatives to address shortage of nurses include importing skills, as was used in the Western Cape ICUs of two private hospital groups, and using retired nurses in the public sector (Erasmus & Breier 2009:156). In Gauteng Province, a similar initiative was used in a particular public hospital, and it worked well.

Anecdotal observation is that at individual hospital levels, other temporary measures may be used to curb the nursing shortage such as employment of agency nurses, working overtime by permanent staff, or short-term deployment of nursing staff from other units within the hospital. In the settings for this study, deployment from ICU usually happened particularly during the times when the units had an extra nurse on duty.

## Problem statement

In these particular ICUs, the nurse staffing was planned according to the number of patients, and often there was a *floating* nurse. The *floating* nurse was usually an extra registered nurse who was on standby for new admissions (planned or unplanned) and emergencies, and was also available to assist other ICU nurses with patient care and other activities in the unit. A *floating* nurse may also be one who may not have a patient at any given time; be it because the patient has gone for a procedure out of the unit, his or her patient has been discharged out of ICU, or the patient has demised. Different ICU nurses happened to assume the roles of *floating* nurses on different days, and therefore not only one nurse was always deployed.

According to the observation in this study, when there is an extra nurse in ICU the ICU manager at the time is often ordered to deploy that nurse to other units where there is a shortage. The units did not have policies on redeployment nor a protocol that indicates that if a nurse is on duty in ICU and does not have a patient, for whatever reason, she or he should then be sent to other units where there is shortage of staff. Thus, if the nurse without a patient happened to be an agency nurse or a nurse doing overtime, she or he would rather be sent home, except if it was during the night. If the nurse happened to be a permanent staff member in the unit, she or he would be sent to other units where there was a need for extra staff. Deployment from ICU to other units is practiced, but it is never nurses from other units who are deployed to ICU. This is because nurses from the general wards and other units, such as neonatal ICU, reportedly do not have experience in working in the adult ICUs.

The argument in this article is that nurses in South Africa have undergone general or comprehensive training. However, each one of them has developed expertise in the field of nursing where they are working. The assumption is that management often does not consider the ICU nurses’ expertise before implementing short-term deployment to other units. With regard to deployment, there is no consultation rather only instruction. Deployment can be a stressful event, depending on the area of allocation. It is therefore necessary to understand the views of ICU nurses regarding short-term deployment to other units.

## Purpose of the study

The purpose of this study was to explore the views of ICU nurses regarding short-term deployment to other units.

## Research method and design

A qualitative, exploratory design was used (Denzin & Lincoln 2011) following interpretivism (Creswell 2009). The qualitative approach was used because the researcher needed to obtain information from the ICU nurses by exploring their views regarding their deployment to other units (Burns et al. 2012). The theoretical assumption in this study was that qualitative research is used in order to understand the ICU nurses’ encounters and the meanings they assigned to deployment, as this was necessary for the generation of narrative data. The researcher conveyed an understanding of the views by reporting the realities in detail through presentation of themes that reflect the descriptions by the participants.

The setting was a 20 bed multidisciplinary level I ICU of a public and academic hospital and a 14 bed level II ICU of a private hospital in Gauteng Province, South Africa. The average bed occupancy in these units was 12. However, the units could have more or fewer patients depending on patient flow at different times. The units were both adult ICUs. The ICUs of these hospitals were selected because short-term deployment of nurses to other units was practiced.

The population was the ICUs nurses. Purposive sampling was used to select the participants. To be included in the study the participant had to be a registered nurse, ICU trained or non ICU trained, with experience of one or more years working in the ICU, and currently working in the specific ICUs selected for the study.

### Data collection and analysis

Two focus group interviews (FGIs) were conducted. The interviews were audio recorded with the permission of the participants. The focus groups consisted of 6 and 7 participants each who were included in the interviews according to their availability and willingness to participate. The participants were all female with experience of 2 to 18 years working in the ICUs. The researcher was the moderator during the focus group. The FGIs took place in the ICUs of the two selected hospitals and lasted 25 minutes each. Audio recorded interviews were transcribed verbatim and data were analysed manually (Creswell 2012) using open coding.

## Ethical considerations

Ethical approval was obtained from the Higher Degrees Committee of the Department of Health Studies, UNISA. Permission to conduct the study was obtained from the Department of Health, Gauteng Province and the management of each hospital through their ethical committees, who were provided with the relevant documentation from the researchers. A written consent form was signed by the participants following a thorough explanation of the purpose of the study. The participants were treated as autonomous agents by allowing them voluntary participation in the study. Privacy and confidentiality were maintained in that their names did not appear in the audio records and transcripts (Polit & Beck 2008:170). Only code numbers and dates of data collection were used to identify the audio tapes and transcripts. The participants were assured that they had the right to withdraw from the interviews without penalty (Burns et al. 2012). No person was coerced, or to some degree forced, to participate. The consent forms were kept safely and separate from the transcripts.

## Trustworthiness

Guba’s model of trustworthiness criteria (Lincoln & Guba 1985, quoted in Polit & Beck 2008:539) was used to ensure trustworthiness. Credibility was achieved through prolonged engagement with the participants. Amongst the focus groups were the registered nurses who were once deployed to other units in order to ensure that data were consistent and believable. On the spot member check with the participants was done to confirm the accuracy of data captured, and the meanings that the ICU nurses wished to impart regarding deployment from ICUs to other units. A short poster presentation was made to the participants as a follow up to share the findings with the participants. Two experts in qualitative research were asked to review the research plan and implementation and to verify the data analysis (Krefting 1991). An audit trail of the methods was kept to ensure objectivity of the study. The findings of this study are transferable to the ICUs of the hospitals that were included in this study (Polit & Beck 2008:539).

## Results

Three themes that emerged from the views of the ICU nurses regarding short-term deployment were consultation and negotiation, recognition of ICU nurses expertise and the need for a deployment policy and procedure.

### Consultation and negotiation

The participants were of a view that the ICU nurses needed to be consulted before being deployed to the other units. Again, negotiation should be done with the unit manager rather than instruct her or him to deploy the nurse.

With regard to negotiation, the participants were of a view that there is a lack of autonomy when it comes to management of staffing the unit, because the ICU manager was not given full autonomy to manage their staff in the unit:

‘As it is of right now, she [the unit manager] is here but they dictate to her. Most of the time she does not have the power to say.’

### Recognition of intensive care unit nurses expertise

The participants were of a view that deployment brings about problems with recognition of expertise in the area of allocation. According to the participants, the fact that they were working in ICU meant that they were experts in ICU and therefore could be left to work in their respective units:

‘For instance we are regarded as intensivists neh?’

‘But then you will find a situation where we are like being sent out to the entire hospital.’

In relation to the lack of recognition of expertise, the view of the participants was that this leads to improper utilisation of staff as they are deployed anywhere in the hospital without consideration of their abilities to perform. The findings indicated that the participants were not confident to work in new units for a brief period as there was no time for orientation. Some participants said:

‘For the mere fact that we are from ICU, they redeploy us. This is improper utilization of staff.’

‘For instance they do not take into consideration whether I am experienced in that unit.’

‘Lying-in wards, it is an area where really people have long lost touch with such layouts.’

The participants further acknowledged the risks of being placed in the units where they had lost touch with current practice:

‘Sometimes we are even posted to areas where we have long forgotten to function in a unit like that. For instance neonatal ICU is a specialty on its own as compared to general ICU.’

### The need for a deployment policy and procedure

The participants were of the opinion that short-term deployment policies and procedures were necessary to ensure fairness in the practice of deployment:

‘It is only ICU nurses to other units, but it can never be vice versa, which is unfair.’

‘But sometimes when we are quite then the staff in the ICU is posted to various departments which is really not fair.’

## Discussion

The purpose of this study was to explore the views of ICU nurses regarding short-term deployment of ICU nurses where short-term deployment is practiced. A non-formal relationship develops between the manager and the subordinates which includes actions, such as giving instructions (Bezuidenhout, Garbers & Potgieter 2007:3). The instructions are mostly influenced by the facets of the work environment and, in this study, could include middle managers instructing the ICU operational manager to deploy ICU nurses to other units. The findings indicated that the participants were concerned with short-term deployment. Whilst this was not a comparative study, the views related to deployment were similar in both public and private hospital ICUs for this study.

The practice of the *floating* nurse being moved from one unit to the other, or so-called redeployment, especially during shortage of staff in certain units, is a big problem for the ICU nurses. Interestingly, this seems not to be a new phenomenon because in Los Angeles (LA) in 2003, 3000 nurses went on strike in protest against this kind of practice (Lindemann & McAthie 1990:52). The ICU nurses felt that they were obliged to heed the deployment call because of fear of insubordination. Bezuidenhout et al. (2007:71) indicate that the specific duties of the employees towards management or employer are to perform diligently and competently and to obey all lawful and reasonable instructions of the employer. However, in this study, senior hospital managers seemed to take advantage of this implied duty that the ICU managers are under the obligation to deploy nurses when instructed to do so.

Unfortunately, there were no policies regarding deployment of staff. Therefore, deployment was viewed as a challenge to the ICU managers with regard to autonomy in management of the nursing staff in their units, and the expertise of the ICU nurses in relation to working in other units. Bezuidenhout et al. (2007:4) mentioned, in addition, that nursing management is perceived to be autocratic in its actions, where senior managers are used to making snap decisions, issuing instructions and also maintaining control without considering the views or wishes of the subordinates. Hass, Coyer and Theobald (2006:145) argued that one way of becoming more effective at work and to increase autonomy is to develop trusting relationships amongst staff.

One of the typical provisions in the employment contract is the job. The Basic Conditions of *Employment Act* (South Africa 1997) regulates the provisions for minimum terms of employment. This should have been taken into account when it came to deployment of the ICU nurses.

With regards to recognition of expertise, the participants indicated that they felt that they should be properly utilised as they are ICU trained and or experienced. Every nurse working in a new or different area needs to be orientated. On the other hand, the participants were of the opinion that there is a tendency to think they can work anywhere, not considering the challenges that they may encounter. Some of the challenges indicated with deployment were lack of, or no, orientation since the nurse would work for a brief period, and also because of the need to immediately perform the routine and nursing duties for the shift. In addition, there was no time to orientate the deployed nurse due to shortage of staff and also fear of delaying routine work. Over and above this, there were challenges with regard to unfamiliar layout and design of the unit which had a negative impact on the delivery of nursing care. Without orientation the nurse’s performance may be counterproductive.

Hass et al. (2006:9) indicate that familiarity with the environment will contribute to a more balanced approach to care. Nurses have been trained generally. However, each one of them develops expertise in the field of nursing where they are working. For a nurse to function effectively and efficiently in a new unit, she or he needs to know the layout of that unit, have up to date knowledge and skills, expertise to function, and be orientated. Lack of the above-mentioned can lead to feelings of inadequacy and deskilling.

The implications of taking a nurse out of ICU and placing her or him in a different unit may have consequences to the patient’s needs, to the unit systems and functioning and the deployed nurse’s skills. Against this background, it is understandable that ICU nurses should feel frustrated by the lack of orientation during deployment.

It is good management practice to undertake periodic reviews of nurse staffing views. Decisions regarding deployment should be informed by detailed knowledge about a particular ward or department. Once made, deployment should be monitored for its impact on patient and nurses’ outcomes.

It is evident in this study that the participants need to have a say over deployment, especially because there are no written policies or guidelines regarding deployment. Besides, there is limited literature on ICU nurses’ deployment in South Africa, except for studies of deployment in the military services.

## Limitations of the study

Only two hospital ICUs that practiced redeployment were included in this study. This limits the application of the findings to a wider range of ICUs or even other hospitals in the rest of South Africa.

### Recommendations for nursing practice and future research

This article presents to the reader an understanding of the context of short-term deployment to the different unit from ICUs. Deployment policy and adherence to minimum conditions of service is essential in nursing practice. Policies regarding redeployment should be designed that suit the specialty areas such as ICU. Senior managers should recognise the feeling of ICU nurses towards deployment to other units. Proper consultation and negotiation is necessary prior to deployment. Further research is essential in other units practicing short-term deployment of staff and into the experiences of staff working in the units where ICU nurses have been deployed.

## Conclusion

Short-term deployment may impact negatively on the patients’ needs due to the staff expertise. Consultation and negotiation is essential prior to the deployment of staff. Management should recognise and acknowledge the expertise of ICU nurses in their own specialty area.
